# An Assessment of the Degradation Potential and Genomic Insights Towards Hydroxylated Biphenyls by *Rhodococcus opacus* Strain KT112-7

**DOI:** 10.2174/0113892029319746240812051356

**Published:** 2024-08-21

**Authors:** Darya Egorova, Bjorn Olsson, Tatyana Kir’yanova, Elena Plotnikova

**Affiliations:** 1 Laboratory of Microbiology of Technogenic Ecosystems, Institute of Ecology and Genetics of Microorganism, UB RAS, Perm, Russia;; 2 School of Biosciences, University of Skövde, Skövde, Sweden

**Keywords:** *Rhodococcus opacus*, hydroxylated biphenyls, *bph* genes, degradation, plasmid, metabolic pathway

## Abstract

**Background:**

Hydroxylated biphenyls are currently recognized as secondary pollutants that are hazardous to animals and humans. Bacterial degradation is the most effective method for the degradation of hydroxylated biphenyls. Several strains capable of degrading polychlorinated biphenyls have been described, which also degrade hydroxylated biphenyls.

**Objectives:**

1) To study the biodegradative properties of the *Rhodococcus opacus* strain KT112-7 towards mono-hydroxylated biphenyls. 2) To analyze the genome of the *Rhodococcus opacus* strain KT112-7. 3) To identify the genetic basis for the unique biodegradative potential of the *Rhodococcus opacus* strain KT112-7.

**Methods:**

A genome analysis of the strain KT112-7 was conducted based on whole-genome sequencing using various programs and databases (Velvet, CONTIGuator, RAST, KEGG) for annotation and identification of protein-coding sequences. The strain KT112-7 was cultivated in a K1 mineral medium supplemented with mono-hydroxy biphenyls or mono-hydroxybenzoic acids as the carbon source. For the growth test mono-hydroxybiphenyls or mono-hydroxybenzoic acids were dosed at concentrations of 0.5 g/L and 1.0 g/L correspondently, and the bacterial growth was monitored by the optical density. For the biodegradative activity test, mono-hydroxybiphenyls were dosed at a concentration of 0.1 g/L in vials, inoculated with late exponential phase bacteria previously acclimated on biphenyl. Compound analysis was performed using GC-MS, HPLC, and spectrophotometry.

**Results:**

It was found that the genome of strain KT112-7 consists of a chromosome and 2 plasmids. Biphenyl degradation genes (*bph* genes) were identified on plasmid PRHWK1 and the chromosome, as well as hydroxybenzoic acid degradation genes on the chromosome. The strain KT112-7 was shown to degrade mono-hydroxylated biphenyls to basal metabolic compounds of the cell, with the highest destructive activity observed towards 3- and 4-hydroxylated biphenyls (98%).

**Discussion:**

Analysis of the translated sequences of the *bph* genes from strain KT112-7 revealed that the amino acid sequences of the *bph* operon, located on plasmid pRHWK1, exhibit high similarity to homologous enzymes of the "upper" pathway for biphenyl degradation in Rhodococcus jostii RHA1, whose *bph* genes are also plasmid-borne. The deduced amino acid sequences encoded by the bphA1A2B genes, located on the chromosome of strain KT112-7, show a high degree of similarity to enzymes from Rhodococcus strains that mediate naphthalene degradation. The genes of the "lower" biphenyl pathway in strain KT112-7 are located on the chromosome and share a high level of similarity with the *bph* genes of Acidovorax sp. KKS102. Analysis of the deduced sequences of the pcaGHBCDF, pcaIJfadA, and catABC genes, along with the metabolites identified during the cultivation of strain KT112-7 on hydroxylated biphenyls, suggests the presence of both classical and unique metabolic pathways for hydroxylated benzoic acids in strain KT112-7.

**Conclusion:**

The *Rhodococcus opacus* strain KT112-7 is characterized by genetic systems that contribute to its high biodegradative potential towards mono-hydroxylated biphenyls and their metabolites. Thus, the strain KT112-7 is promising for use in hydroxybiphenyl degradation technologies.

## 1 INTRODUCTION

Within the framework of the concept of “sustainable development” adopted by all developed countries worldwide, the issue of restoring the environment to a safe state for humans is of significant importance. One of the mechanisms for resolving these issues is the adoption of international agreements regulating the use of certain substances. In 2001, the Stockholm Convention on Persistent Organic Pollutants was adopted, according to which a number of compounds and groups of compounds should be phased out of production and destroyed. Polychlorinated biphenyls (PCBs) are included in the list of these compounds (http://chm.pops.int).

The basis of the PCB molecule is a diaryl structure - biphenyl [1]. Biphenyl is found in the environment in petroleum, natural gas, and various resins [2, 3]. Under the influence of biotic factors, biphenyl is transformed into hydroxylated biphenyls, which pose a danger to mammals and the environment as a whole [4-9]. The main metabolites during the degradation of biphenyl in the strain *Pseudomonas* sp. SBUG 2067 are monohydroxybiphenyls [10]. The cytochrome P450 monooxygenase of the strain *Mycobacterium* sp. PYR-1 oxidizes biphenyl to 4-hydroxybiphenyl, which is further metabolized to 4-hydroxybenzoic acid [11]. The transformation of biphenyl into monohydroxylated derivatives also involves o-xylene dioxygenase from the strain *Rhodococcus* sp. DK17, resulting in 2-hydroxy- and 3-hydroxybiphenyl derivatives [12]. Dihydroxylated biphenyls are formed through enzymatic reactions catalyzed by dioxygenase, in particular biphenyl 2,3-dioxygenase [5].

Monohydroxylated biphenyls are formed in the environment as products of biotransformation of polycyclic compounds, including those containing sulfur in their structure [13-17]. The most described pathway is the biodegradation of dibenzothiophene (4S-pathway). 2-Hydroxybiphenyl is the product formed as a result of the transformation of dibenzothiophene by strains *Bacillus aryabhattai* NM1-A2, *Gordonia alkanivorans* 135, *Gordonia alkanivorans* 1B, *Microbacterium* sp. ZD-M2, *Mycobacterium *sp. G3, *Paenibacillus* sp. A11-2, *Rhodococcus erythropolis* SHT87, *Rhodococcus *sp. SL-9, *Rhodococcus erythropolis* D1, *Rhodococcus erythropolis* IGT8, *Tsukamurella paurometabola* 3OW [9, 18-25]. It has been shown that for *Gordonia alkanivorans* 1B and *Rhodococcus erythropolis* D1, 2-hydroxybiphenyl is toxic at concentrations of 0.52 - 0.62 mM under acute exposure and 0.21 - 0.49 mM under chronic exposure, and also exhibits fungicidal action [9, 26]. The toxic effect of 2-hydroxybiphenyl is caused by enzyme suppression induction [27]. Currently, the strain *Rhodococcus erythropolis* IGTS8 and the bacterial community IQMJ-5, which degrade dibenzothiophene, are resistant to the negative effects of 2-hydroxybiphenyl and capable of using it in their metabolism [28, 29].

The biotransformation of monohydroxylated biphenyls, particularly 2-hydroxybiphenyl, occurs through the oxidation pathway involving monooxygenase enzymes. The oxidation pathway of 2-hydroxybiphenyl under the action of 2-hydroxybiphenyl 3-monooxygenase is most extensively described [26, 30-33]. As a result of oxidation at the 3-carbon atom of the hydroxyl-substituted ring of 2-hydroxybiphenyl, 2,3-dihydroxybiphenyl is formed, which is a substrate for 2,3-dihydroxybiphenyl 1,2-dioxygenase, the “upper” biphenyl pathway enzyme in aerobic bacteria [5, 34-36]. Additionally, the transformation of 2-hydroxybiphenyl into 2-methoxybiphenyl has been demonstrated in the strain *Achromobacter* sp. NBTU-02 [37].

As a result of chemical synthesis based on biphenyl, polychlorinated biphenyls (PCB) were obtained. PCBs were produced and used on an industrial scale for several decades [38, 39]. However, at present, the hydroxylated derivatives of PCBs are formed from them under the influence of climatic factors and physiological processes in biological organisms. Several studies from 2004 to 2022 have shown the presence of hydroxylated PCBs in the organisms of marine mammals, polar bears, birds that consume fish, domestic animals, and humans [40]. It is suggested that hydroxylated PCBs are formed both as a result of PCB oxidation in organisms under the action of the cytochrome P450 system and in the atmosphere during the reaction of PCBs in the gas phase with hydroxyl radical [11, 12, 41, 42]. There is evidence of the negative impact of modified PCBs (particularly those containing a hydroxyl group) on the reproductive system of fish and the feminization of embryos [43]. Thus, hydroxylated PCBs have now acquired the status of secondary pollutants. There are some publications on hydroxy-PCB degradation by aerobic bacteria possessing biodegradative activity towards biphenyl/PCB [30, 33, 37, 44-47]. These studies have shown the ability of aerobic bacteria to degrade hydroxylated PCBs using both biphenyl degradation enzymes (the “upper” and “lower” pathways) and monooxygenase class enzymes.

In our previous studies, it was established that *Rhodococcus opacus* strain KT112-7) has a degradation potential for a wide range of aromatic compounds, including PCBs and their hydroxylated derivatives [46-49]. It has been shown that the *Rhodococcus opacus* strain KT112-7 accomplishes the destruction of hydroxylated derivatives of commercial mixtures of PСB under the trade names Sovol and Trichlorobiphenyl (analogues of Aroclor 1254 and Aroclor 1242/Delor 103 respectively) by 96.9–100% in 10-14 days at an initial concentration of 0.1 g/L [46, 47]. The efficiency of destruction of individual congeneric groups in the hydroxy derivative mixture Delor 103 at an initial concentration of 0.01 g/l by the strain *Pseudomonas* sp. P1B16 ranged from 5 to 100% [33]. The metabolic pathway of transformation of 3-hydroxy-4-chlorobiphenyl and 3-chloro-4-hydroxybiphenyl by strain KT112-7 was further investigated [50]. It was established that the molecule oxidation of these compounds occurs on the unsubstituted ring under biphenyl 2,3-dioxygenase, resulting in the formation of monochlorinated monohydroxylated benzoic acids. The biodegradation of 4-hydroxy-3-chlorobiphenyl is described for the strain *Sphyngomonas* sp. N-9 (100% at an initial concentration of 0.1 g/l) [51]. These data suggest that the strain KT112-7 has biodegradative potential towards monohydroxylated biphenyls. However, the genetic basis of the biodegradative properties of this strain remains unclear, as well as the catabolic pathway of mono-hydroxylated biphenyls. Therefore, this study aims to investigate the biodegradative properties of the *Rhodococcus opacus* strain KT112-7 towards mono-hydroxylated biphenyls, analyze the genome of the strain, and uncover the genetic determinants of its unique biodegradative potential.

## 2 MATERIALS AND METHODS

### 2.1 Chemicals

2-hydroxybiphenyl (2HO-biphenyl), 3-hydroxybiphenyl (3HO-biphenyl), 4-hydroxybiphenyl (4HO-biphenyl), 2-hydroxybenzoic acid (2HBA), 3-hydroxybenzoic acid (3HBA), 4-hydroxybenzoic acid (4HBA) and dihydroxybenzoic acids, catechol (99% purity) were obtained from Sigma-Aldrich (Steinheim, Germany). Unsubstituted biphenyl (99% purity) was obtained from ACROS-organics (New Jersey, USA). Mineral salts and acids (>98% purity) were purchased from ZAO Ekros (Saint Petersburg, Russia) and the solvents for HPLC (HPLC grade) were obtained from OOO NPK Kriokhrom (Saint Petersburg, Russia).

### 2.2 *Rhodococcus opacus* Strain KT112-7

The strain *Rhodococcus opacus* KT112-7 (identified in early publications as *Rhodococcus wratislaviensis* KT112-7 (VKM Ac-2623D)) was isolated from technogenic-mineral formations of a salt mining enterprise located in Berezniki, Perm Krai, Russian Federation. It has been found that strain KT112-7 is capable of degrading a wide range of aromatic and polyaromatic compounds, including polychlorinated biphenyls and their mixtures, as well as chemically modified mixtures of PCBs containing hydroxylated derivatives [46-49].

### 2.3 Degradation of Mono-hydroxybiphenyls

For the investigation of biodegradation of individual congeners of mono-hydroxybiphenyls, the bacterial cultures pre-grown in a K1 mineral medium [52] supplemented with biphenyl (1 g/L) as the carbon source until the middle exponential phase (OD_600_=1.5) were placed in 4 mL Wheaton sample vials (Sigma-Aldrich, Germany) closed with PTFE-lined stoppers. The vials containing bacterial cultures without biphenyl were individually dosed with 2-hydroxy-, 3-hydroxy-, and 4-hydroxybiphenyls (0.1 g/L) and incubated on a shaker (Biosan ES-20/60, Riga, Latvia) at 120 rpm, 28°C for 3 days. The samples for analysis were taken at 0, 1, and 3 days of incubation. The destruction process was stopped by freezing. Extraction and analysis of hydroxybiphenyls were performed by GC-MS, and metabolites were analyzed by HPLS, as described [53].

### 2.4 The Growth Test of the Strain KT112-7 on the Biphenyl, Mono-hydroxybiphenyls or Mono-hydroxybenzoic Acids

The strain KT112-7 was cultivated in a periodic mode in 100 mL of K1 mineral medium in 250 mL Erlenmeyer flasks on a rotary shaker (Biosan ES-20/60, Riga, Latvia) at 120 rpm and 25°C. For the growth test, biphenyl, mono-hydroxybiphenyls or mono-hydroxybenzoic acids were dosed at concentrations of 1.0 g/L, 0.5 g/L, and 1.0 g/L correspondently and were used as carbon sources. Periodic cultivation was carried out for 7 days. The bacterial growth was monitored by measuring the optical density at 600 nm (Biospec-mini, Shimadzu, Japan).

### 2.5 Detection of the Mono-hydroxybiphenyls and their Degradation Products

The concentrations of mono-hydroxybiphenyls were performed by GC-MS, and metabolites were analysed by HPLC [49, 53].

Quantitative and qualitative analysis of hydroxybiphenyls was performed using gas-chromato-mass-spectrometer Agilent GC 6890N MSD 5973N with quartz capillary column HP-5MS SN US15189741-1 (60 m x 0.25 mm) (“Agilent Technology,” USA). The initial column temperature was 40°С (3 min), followed by temperature increase at a rate of 10°/min up to the final temperature of 290°С (isotherm for 30 min). The evaporator temperature was 250°С, the quadrupole temperature was 150°С, and the transition chamber temperature was 280°С. The volume of the injected sample was 1 µl.

Moreover, to perform analysis in a flask containing water cultural medium, a mixture of concentrated Н_2_SO_4_ −12.5% SDS-hexane (1:10:25) was added, and extraction was carried out for 60 min at 30^°^С. For better phase separation, the flasks with extractant were subjected to centrifuging for 5 min using a Sigma 3K30 (Sigma, Germany) centrifuge at a rate of 10,000 rpm and dehydrated with Na_2_SO_4_. Then, an organic layer was analyzed.

The calculation of hydroxybiphenyl content in every sample was performed by internal normalization. Based on the calculated squires of the peaks, the contents of hydroxybiphenyls were estimated after the biodegradation process.

The presence of HBAs in the supernatant was accomplished using chromatography LC-20A (“Shimadzu”, Japan) with Discovery C18 column (150 x 4.6 mm or 250 х 4.6 mm) (“Supelco”, “Sigma-Aldrich”, USA) и UV-detector at 205 nm. An analysis was performed using acetonitryl-0.1% Н_3_РО_4_ (70:30). The identification was performed by comparing retention time values of standard and tested compounds. The amount of the products formed was estimated by the area and height of the peaks compared to those of the standard products.

### 2.6 Calculation of Kinetic Growth Parameters and Substrate Utilization

The efficiency of hydroxybiphenyl degradation was evaluated as a percentage using the formula:

D (%) = (C_t_ × 100) / C_ 0 _ (1),

where D is the efficiency of degradation (%), Ct is the concentration of hydroxybiphenyls at a certain time interval (2 or 24 h), and C_0 _ is the initial concentration of chlorobiphenyl [47].

The specific rate of hydroxybiphenyl and hydroxybenzoic acid degradation (V_t_) was calculated using the formula:

V_t_ = (C_0 _ - C_t_) / t (2),

where C_0 _ is the initial concentration of hydroxybiphenyls and hydroxybenzoic acids, C_t_ is the concentration after 7 days of the experiment, and t is the destruction time (days) [46].

The maximal specific growth rate of strain KT112-7 (μ) was determined using the equation:

μ = (1n X_t_ - 1n X0) / (t - t) (3),

where X is the optical density (OD_600_) and t is the time (days) [54].

### 2.7 Genomic DNA Preparation

For DNA extraction, the culture of strain KT112-7 in the middle exponential growth phase was centrifuged at 11,000 × g using a 3K30 centrifuge (Sartorius, Germany). The obtained pellet was washed from biphenyl in a K1 mineral medium and centrifuged again. The genomic DNA was extracted from bacterial biomass using the SDS-CTAB method [55].

### 2.8 Genome Sequencing and Analyses

The draft genome of the strain KT112-7 was obtained at the GenoAnalytica company, Moscow, Russia (https://www.genoanalytica.ru), using the Illumina HiSeq 1500 platform following the manufacturer's protocols. The Illumina data was assembled using Velvet [56], followed by ordering the contigs and identifying plasmids using CONTIGuator [57]. The genomes were annotated using Prokka 1.14.6 [58] with the Barrnap 0.9 plugin and the JGI Microbial Genome Annotation Pipeline [59]. Protein-coding and RNA genes were predicted using RAST (https://rast.nmpdr.org/) [60] and annotated using the Kyoto Encyclopedia of Genes and Genomes (KEGG) database [61]. Homologous sequences were searched in the GenBank database (http://www.ncbi.nlm.nih.gov).

### 2.9 Analysis of Metabolic Pathways for the Degradation of Mono-hydroxybiphenyls and Mono-hydroxybenzoic Acid

The metabolic pathways of hydroxylated biphenyls and hydroxybenzoic acids were analyzed based on experimental data on metabolic profiling, genome analysis of the strain KT112-7, and databases such as BRENDA (http://www.brenda-enzymes.info), KEGG (http://www.genome.jp), and GenBank (http://www.ncbi.nlm.nih.gov). Compound visualization and transformation schemes were performed using the ACDLabs Freeware software.

### 2.10 Modeling the Structure of BphA1 and Docking

Biphenyl dioxygenase (BphA) is a key enzyme in the biodegradation of biphenyl and its derivatives. The α-subunit of biphenyl dioxygenase (BphA1) is the key structure responsible for attaching the enzyme to the substrate. Therefore, in this study, we examined the possible structure of BphA1_KT112-7_. The deductive amino acid sequence of BphA1 from the strain *R. opacus* KT112-7 was obtained based on the nucleotide sequence of the *bph*A1 gene located on the plasmid pRHWK1, which was identified during analysis of the whole genome sequence using RAST (https://rast.nmpdr.org/). The program Modeller 10.2 (https://salilab.org/modeller), a web server for structural bioinformatics called SwissModel (https://swissmodel.expasy.org), and the program UCSF Chimera 1.16 (https://www.cgl.ucsf.edu/chimera) were used for constructing the protein's tertiary structure and visualization, respectively. The templates were searched for in the Protein Data Bank (PDB) (https://www.rcsb.org) using BLAST. Furthermore, the Swiss Model was used for target-template alignment. Protein sequences with the highest identity were selected for analysis. The positions of ligands (2-hydroxy-, 3-hydroxy-, and 4-hydroxybiphenyl) were calculated using http://www.swissdock.ch/. Models of monohydroxybiphenyls (CHEBI:17043 – biphenyl-2-ol, CHEBI:34338 – biphenyl-3-ol, and CHEBI:34422– biphenyl-4-ol) for docking were taken from the website https://www.ebi.ac.uk/chebi/init.do. The clusters were identified using the Chimera 1.16 program (https://www.cgl.ucsf.edu/chimera). The visualization was performed using PyMOL version 2.5.4 (https://pymol.org). Moreover, to control the orientation of the 3D model in space, we used a list of commands from the website https://bionerdnotes.wordpress.com/2018/11/12/getting-high-quality-pictures-in-pymol/.

### 2.11 Statistical Analysis

All the experiments were conducted in triplicate. The inhibition of *Rhodococcus*-strains was modeled according to a first-order inactivation model using the linear regression procedure in MS Office Excel 2013 and STATISTICA 6.0 (http://statsoft.ru). The pearson’s correlation coefficient (***r***) and other statistical indicators were calculated using Excel 2013 and STATISTICA 6.0.

## 3 RESULTS AND DISCUSSION

### 3.1 Kinetics of Hydroxybiphenyl Degradation and Metabolites Identification

**Fig. (1):**
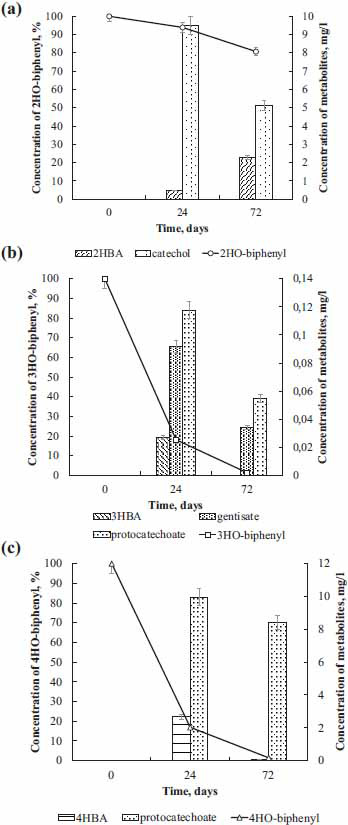
(**A-C**) Degradation of the mono-hydroxylated biphenyls by the strain KT112-7.

In experiments with resting cells, it was found that the strain KT112-7 carries out the degradation of mono-hydroxylated biphenyls (Fig. **1**). The efficiency of degradation was as follows: 2HO-biphenyl - 19.3%, 3HO-biphenyl - 98.7%, 4HO-biphenyl - 98.6%. The low efficiency of 2-hydroxybiphenyl degradation may be due to the negative effect of this compound and its metabolites on the enzymes of bacterial cells [9, 27, 62-64]. During the degradation of 2HO-biphenyl, the main metabolites formed are 2-hydroxybenzoic acid and catechol (Fig. **1A**). Considering the information about metabolic processes in aerobic bacteria, it can be assumed that catechol is formed as a result of dioxygenation of 2HBA and is the main intermediate in its metabolism. During the degradation of 3HO-biphenyl in the culture medium, 3HO-benzoic acid, 2,5-dihydroxybenzoic acid (gentisate), and 3,4-dihydroxybenzoic acid (protocatechuate) were detected (Fig. **1B**). It was worth noting that 3HBA is not detected in the medium after 72 h of incubation, while the concentration of gentisate and protocatechuate were 0.034 ± 0.002 mg/L and 0.058 ± 0.001 mg/L, respectively. Taking into account the fact that the concentration of 3HO-biphenyl after 72 h of incubation is 1.3%, and the analysis of metabolic pathways of bacterial degradation of aromatic pollutants shows that gentisate and protocatechuate are transformation products of 3HBA, it can be assumed that the degradation of 3HO-biphenyl by the strain KT112-7 occurs before the compounds of the basal metabolic compounds of the cell. The degradation of 4HO-biphenyl by the strain KT112-7 is accompanied by the appearance of 4HBA and 3,4-dihydroxybenzoic acid (protocatechuate) in the culture medium (Fig. **1C**). Analysis of the dynamics of the concentration of these compounds shows that the strain KT112-7 effectively decomposes not only the initial compounds (4HO-biphenyl) but also the metabolites formed during this process. An analysis of the metabolic profile during the transformation of mono-hydroxylated biphenyls by the strain KT112-7 suggests that the oxidation of this substrate occurs through the classical pathway of biphenyl degradation.

The ability to degrade mono-hydroxybiphenyls has been described for strains *Microbacterium* sp. ZD-M2, *Mycobacterium* sp. PYR-1, *Pseudomonas* sp. CB-3, and *Pseudomonas* sp. P1B16 [11, 23, 33, 35]. It should be noted that the strain *Microbacterium* sp. ZD-M2 grew in the presence of low concentrations of 2-hydroxybiphenyl (up to 0.1 mM) while increasing the concentration led to inhibition of growth processes [23]. On the contrary, the strain *Pseudomonas* sp. P1B16 effectively degraded 2HO-biphenyl and showed low activity towards 3HO- and 4HO-biphenyls [33]. It is likely that differences in the metabolic activity towards mono-hydroxylated biphenyls between strain KT112-7 and strain P1B16 are determined by the functioning of enzymes from different classes. In the study by Suman *et al.* [33], it was shown that the initial attack on 2HO-biphenyl is carried out by 2-hydroxybiphenyl 3-monooxygenase, while we assume that in the strain KT112-7, biphenyl 2,3-dioxygenase is responsible for the initial attack on the hydroxybiphenyl molecule.

**Fig. (2):**
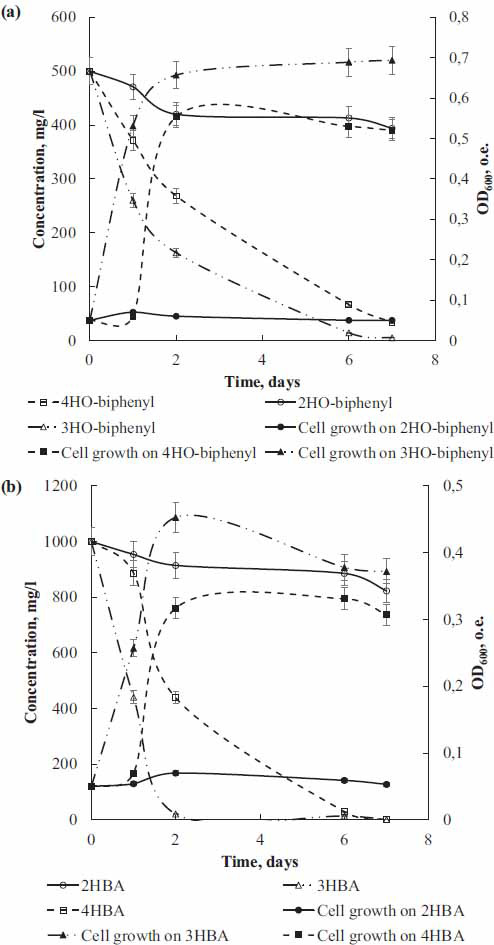
Growth of the strain KT112-7 on mono-hydroxybiphenyl (**a**) and mono-hydroxybenzoates (**b**) and the change in the substrates.

Additionally, to investigate the possibility of using hydroxybiphenyls as a growth substrate for the strain KT112-7, experiments on periodic cultivation in mineral medium with mono-hydroxybiphenyl (Fig. **2a**) or mono-hydroxybenzoic acids (Fig. **2b**) as the sole carbon source for 7 days were conducted. It was found that the strain KT112-7 effectively grows on *meta*- and *para*-mono-hydroxylated biphenyls and benzoates, while growth on 2HO-biphenyl and 2HBA showed minimal increase in optical density of the culture and changes in substrate concentration. The specific growth rate of the strain KT112-7 during cultivation on *ortho*-hydroxylated substrates is an order of magnitude lower than on the others (Table **1**). It has been previously established that actinobacteria, including strains of the genus *Rhodococcus*, grow effectively on mono- and dihydroxybenzoic acids [65]. The analysis of growth parameters showed that the specific growth rate of the strain KT112-7 is higher than that of the strain *R. ruber* P25 during cultivation on 4-hydroxybenzoate, but lower than the specific growth rate of the strain *Microbacterium* sp. B51 on this substrate [65]. Similar to the studies [66, 67], it was noted that the degradation of 4-hydroxybenzoate and the increase in the cell growth of the strain KT112-7 are interrelated. The highest specific destruction rates were found during the growth of the strain KT112-7 on *meta*-hydroxylated biphenyl and benzoate (Table **1**). A strong inverse linear correlation was found between the increase in optical density of the strain KT112-7 culture and changes in substrate concentration (Pearson correlation coefficient ranged from 0.843 to 0.976), indicating the utilization of mono-hydroxybiphenyls and mono-hydroxybenzoates by the strain KT112-7 as a carbon source.

### 3.2 General Genome Features of the Strain КТ112-7

The KT112-7 genome consists of three replicons: a chromosome of 7,587,912 bp, megaplasmid pRHWK1 of 281,912 bp, and megaplasmid pRHWK2 of 130,937 bp (Table **S1**). The chromosome has a GC-content of 67.5%, whereas the GC-content for megaplasmids is 64.3 and 64.1. In total, 7931 protein-coding sequences were predicted, with 7554 located on the chromosome, 326 on the megaplasmid pRHWK1, and 160 on the megaplasmid pRHWK2 (Table **S1**). The additional genome statistics and the distribution of the genes into functional categories are presented in Fig. (**S1**).

The genomic sequences were deposited in GenBank as CP072193.1 – chromosome KT112-7, CP072194.1 - megaplasmid pRHWK1, and CP072195.1 - megaplasmid pRHWK2.

In our early studies, analysis of the nucleotide sequence of the 16S rRNA gene of strain KT112-7 showed 100% similarity to the 16S rRNA gene of the *Rhodococcus wratislaviensis* strain NCIMB 13082^T^ (GenBank Z37138) [48]. In the present study, we performed a quality control test by average nucleotide identity (ANI) to ensure correct strain identification [68, 69]. The results showed that the genome sequences of strain KT112-7 are 99.294% identical with ANI to the type genome of* Rhodococcus opacus* DSM 43205^T ^(GenBank GCA_910591545.1), with 88.9% coverage of the genome.

### 3.3 Analysis of Genes and Enzymes Involved in Hydroxybiphenyl Degradation

As shown in section 3.1, strain KT112-7 could degrade monohydroxylated biphenyls to monohydroxibenzoic acid (Figs. **1** and **2**, Table **1**). In addition, to better understand the possible metabolic process, the related genes were analyzed.

**Fig. (3):**
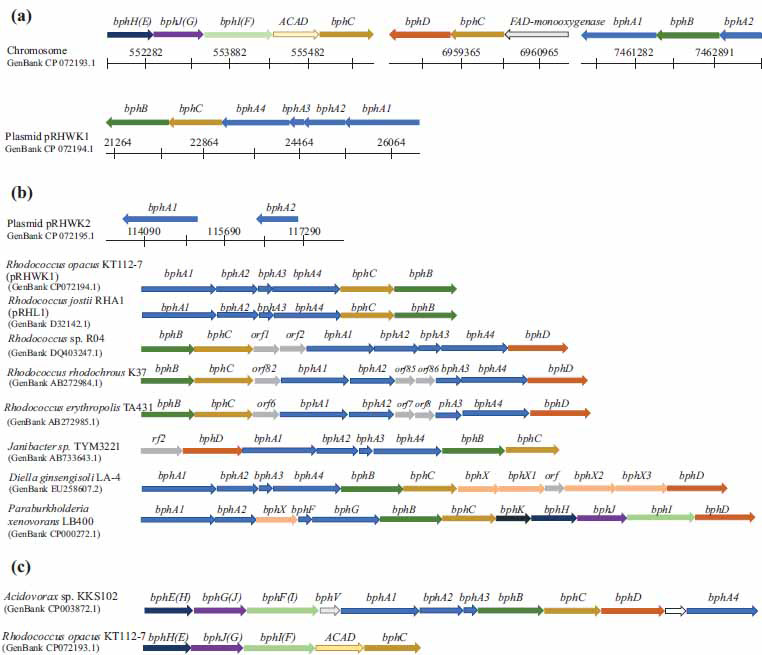
The organization of gene clusters encoding biphenyl degradation enzymes in the *R. opacus* KT112-7 (**a**). Comparison of “upper” *bph*-cluster (**b**) and “lower” *bph*-cluster (**c**) of the strain KT112-7 with those of biphenyl/PCB degrading strains. The description of genes is presented in section 3.3.

It has been well-established that the biphenyl pathway (“upper” and “lower”) is the main one in microbial degradation of biphenyl and its derivatives [4]. The analysis of the complete genome of the strain KT112-7 revealed the presence of genes/enzymes involved in the “upper” and “lower” pathways of biphenyl degradation (Fig. **3**). It was found that the genes of the biphenyl pathway are arranged in three operons on the chromosome (*bphEGFC*, *bphCD* FAD-monooxygenase, *bphA1BA2*), with the *bphA1А2А3А4СВ* operon located on the plasmid pRHWK1, and the *bphA1A2* genes located on the plasmid pRHWK2 (Fig. **3a**). An analysis of the arrangement of *bph* genes showed that in the *bphA1А2А3А4СВ* operon (plasmid pRHWK1), the genes are arranged in the same order and have the same size as the *bph* genes of the strain *Rhodococcus jostii* RHA1 (plasmid pRHL1), but differ from the arrangement of *bph* operon genes in other biphenyl-degrading strains of the genera *Rhodococcus*, *Janibacter*, *Diella*, *Cupriavidus*, and *Paraburkholderia* (Fig. **3b**). Interestingly, the *bph*D gene was only found on the chromosome and not on the plasmid of the strain KT112-7. A similar arrangement of the *bph*D gene has been described previously for the strain *Rhodococcus jostii* RHA1 (Fig. **3a**) [69, 70]. It should be noted that the genes of the “lower” biphenyl pathway of the strain KT112-7, located on the chromosome, are arranged in the same sequence as the strain *Acidovorax* sp. KKS102, and are separate from the other *bph* genes (Fig. **3c**) [4, 70-73].

**Fig. (4):**
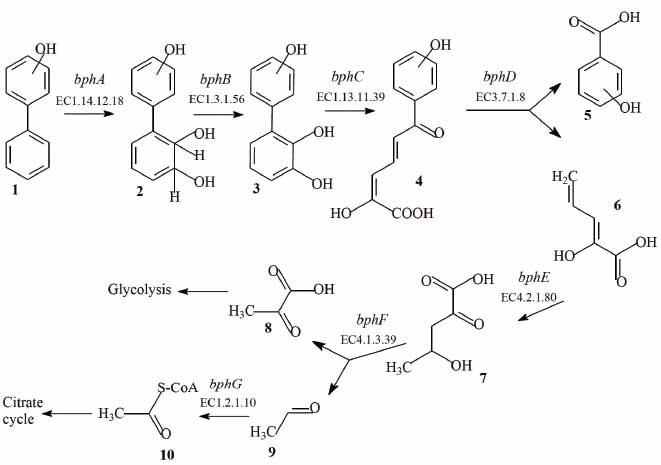
Scheme of the “upper” and “lower” pathways of destruction of mono-hydroxybiphenyls by the *R. opacus* strain KT112-7. 1 – hydroxybiphenyl; 2 – hydroxy-2’,3’-dihydro-dihydroxybiphenyl; 3 – hydroxy-2’,3’-dihydroxybiphenyl; 4 – 2-hydroxy-6-oxo-6-(hydroxyphenyl)-hexa-2,4-dienoate; 5 – hydroxybenzoic acid; 6 – 2-oxopenta-4-enoate; 7 – 4-hydroxy-2-oxovalerate; 8 – pyruvic acid; 9 – acetaldehyde; 10 – acetyl-CoA. The names of genes and numbers of enzymes catalyzing the reaction are indicated above the arrows.

It is hypothesized that the identified genes encode the following enzymes:

“upper” pathway: *bphA1*_chromosome_ (987bp), *bphA1*_pRHWK1_ (1382bp), *bphA1*_pRHWK2_ (984bp) – α-subunit of biphenyl 2,3-dioxygenase; *bphA2*_chromosome_ (519bp), *bphA2*_pRHWK1_ (564bp), *bphA2*_pRHWK2_ (520bp) – β-subunit of biphenyl 2,3-dioxygenase; *bphA3*_pRHWK1_ (324bp) – ferredoxin, *bphA4*_pRHWK1_ (1239bp) – ferredoxin reductase; *bphС*_chromosome_ (912bp, 1134bp), *bphC*_pRHWK1_ (696bp) – 2,3-dihydroxybiphenyl 1,2-dioxygenase; *bphD*_chromosome_ (885bp, 840bp) – HOPDA hydrolase;

“lower” pathway: *bphH(E)*_chromosome_ (786bp), *bphH(E)*_pRHWK1_ (753bp) – 2-keto-4-pentenoate hydratase; bphJ(G) _chromosome_ (903bp) – aldehyde dehydrogenase; *bphI(F)*_chromosome_ (1059bp) – 4-hydroxy-2-oxovalerate aldolase (Fig. **4**, Table **S2**).

Translated nucleotide sequences of the *bph* genes were subjected to BLAST analysis. It was found that the amino acid sequences of the “upper” *bph*-operon, located on the plasmid pRHWK1, had the highest similarity to the amino acid sequences of homologous enzymes from the well-known biphenyl-degrading strain *Rhodococcus jostii* RHA1 (91-100%), as well as enzymes involved in benzene and toluene degradation in aromatic compound-degrading strains *Rhodococcus opacus* B4, *Rhodococcus aetherivorans* I24, *Rhodococcus opacus* 1CP, *Rhodococcus opacus* TKN14 (95-100%) (Table **S2**) [4, 74-78]. The amino acid sequences of the “upper” biphenyl degradation pathway genes, located on the chromosome and on the plasmid pRHWK2, were 96-100% identical to the enzymes involved in naphthalene degradation and other aromatic compounds in *Rhodococcus* strains, including *R. opacus* TKN14 (GenBank BAE53376.1), *Rhodococcus* sp. G10 (GenBank ADM94822.1), *R. opacus* 1CP (GenBank ANS26769.1), *Rhodococcus* sp. P400 (GenBank AAR05106.1, GenBank AAR05107.1), *Rhodococcus* sp. WAY2 (GenBank WP159929237.1), *R. opacus* B4 (GenBank BAH47213.1, GenBank BAH47215.1), *R. ruber* OA1 (GenBank AQW45619.1) (Table **S2**). Such a combination of biphenyl degradation genes in one strain (plasmid localization of an operon with high similarity to classical *bph* genes and chromosomal localization of an operon with high similarity to naphthalene degradation genes) has not been described before. It can be suggested that the diversity of *bph* genes present in the genome of the strain KT112-7 is responsible for its unique degradative properties, specifically its ability to oxidize biphenyl and its hydroxy, methoxy, and polyglycol oxy derivatives [46-49].

The key enzyme of the biphenyl pathway is biphenyl 2,3-dioxygenase, which catalyzes the initial step of the deoxygenation of biphenyl and its derivatives. The catalytic center of the enzyme is located on the *α*-subunit (*bphA1*) [79]. Since the genes of the biphenyl pathway on plasmid pRHWK1 of the strain KT112-7 exhibited the highest similarity to the “classical” *bph* genes, it was hypothesized that the translation products of these genes play a leading role in the transformation of hydroxybiphenyls by the strain KT112-7.

**Fig. (5):**
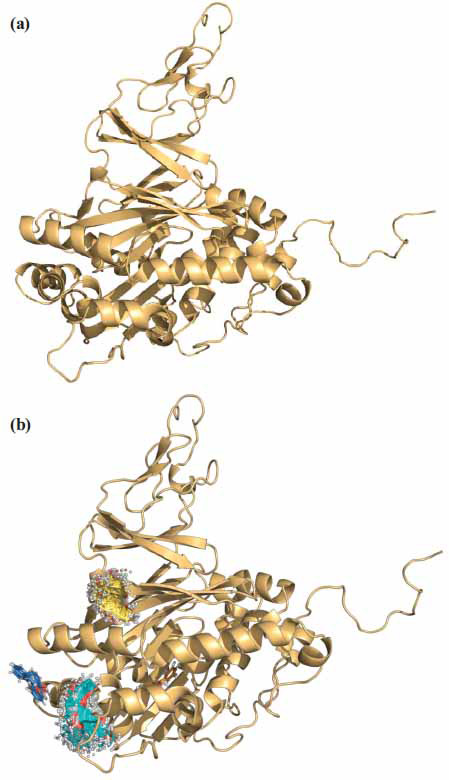
Model of the tertiary structure of the α-subunit of biphenyl 2,3-dioxygenase the strain KT112-7 (**a**) and the probable location of hydroxylated biphenyls during interaction with the enzyme molecule (**b**).

As a result of deductive translation of the nucleotide sequence of the *bphA1*_pRHWK1_ gene and comparison with homologous amino acid sequences presented in GenBank, it was determined that BphA1_pRHWK1_ has the highest level of identity (99.64%) with the primary structure of the *α-*subunit of biphenyl dioxygenase from the strain *R. aetherivorans* I24 (GenBank AAL61663.2). Moreover, using a comparative modeling algorithm from the PDB database, 8 known tertiary structures of *α*-subunits of biphenyl dioxygenases, identified in aromatic compound-degrading strains of *Comamonas*, *Paraburkholderia*, *Pseudomonas*, and *Rhodococcus* genera, were selected based on the identity criterion (>65%) (Table **S3**). The identity of the amino acid sequences of BphA1 from the strain *R. opacus* KT112-7 with the selected enzymes ranged from 65.64% to 98.70%. Based on cluster analysis, taking into account amino acid identity and crystallography as a template for constructing the tertiary structure model of BphA1_pRHWK1_, the *α-*subunit of biphenyl 1,2-dioxygenase from *Rhodococcus jostii* RHA1 (PDB ID: 1ULI_A) was selected (Fig. **S2**, Table **S3**). Based on this sequence, five models were created, and the model with the lowest DOPE score (-51696.81250) was selected as the final model (Fig. **5a**, Table **S4**). BphA1_pRHWK1_ includes 19 *α*-helices and 16 *β*-strands. The surface area of the subunit was 17.99 × 103 Å2, and its volume was 57.81 × 103 Å3. Previously, we obtained a model of the tertiary structure of BphA1_pRHWK1_ using the SwissDock (http://www.swissdock.ch) and SwissModel (http://www.swissmodel.expasy.org) programs [50]. The analysis of the obtained models showed that the use of the Modeller program allowed us to obtain a model of the tertiary structure of BphA1_pRHWK1_ with a higher accuracy score (accuracy score of 0.95, compared to the previous score of 0.93).

It is reported that the efficiency of PCB degradation is influenced not only by the structure of BphA1 but also by the physical, electronic, and geometric characteristics of congeners [80]. It is likely that these parameters may also affect the efficiency of degradation of hydroxylated biphenyl derivatives. An analysis of the possible positions (clusters) of mono-hydroxybiphenyls binding to BphA1_pRHWK1_ was conducted, taking into account energy and structural parameters (Fig. **5b**, Table **S5**). It was found that 2- and 3-hydroxybiphenyls have 35 possible binding clusters, whereas 4-hydroxybiphenyl has 37 clusters. The most optimal position in terms of energy parameters is cluster number 0. This cluster coincides with the catalytic pocket only in the case of 2-hydroxybiphenyl (ΔG = -6.581 J/mol, -6.892 J/mol) (Table **S5**). However, the degradation efficiency of this substrate in the strain KT112-7 is lower compared to 3- and 4-hydroxybiphenyls. It is likely that the inhibition of the expression of enzymatic processes by the 2-hydroxybiphenyl in living organisms is the most significant factor limiting the biodegradation potential of the strain KT112-7 [9, 27]. Analysis of the position of 3- and 4-hydroxybiphenyl clusters on the BphA1pRHWK1 model showed that the best affinity energy are characterized by clusters number 0 (blue area), 1 (green area), and 7 (yellow area) for 3-hydroxybiphenyl (the affinity energy for individual positions was -6.109 J/mol, -6.242 J/mol, -6.290 J/mol) and clusters number 0 (green area) and 1 (yellow area) for 4-hydroxybiphenyl (the affinity energy for individual positions was 6.427 J/mol and 6.210 J/mol) (Fig. **5b**, Table **S5**). These clusters have energy and quantum parameters close to the optimal position, which probably contributes to the effective interaction of biphenyl dioxygenase of the KT112-7 strain with 3- and 4-hydroxybiphenyls and their active oxidation.

### 3.4 Analysis of Genes and Enzymes Involved in Hydroxybenzoic Acid Degradation

The main metabolites of monohydroxybiphenyl degradation in strain KT112-7 are hydroxylated benzoic acids and primarily mono-substituted hydroxybenzoic acids (Figs. **1** and **2**). It is known that aerobic bacteria have several metabolic pathways for the transformation of these. In order to establish the main metabolic mechanisms of the hydroxybenzoic acids in strain KT112-7, genome analysis and a search for the desired genes were carried out.

**Fig. (6):**
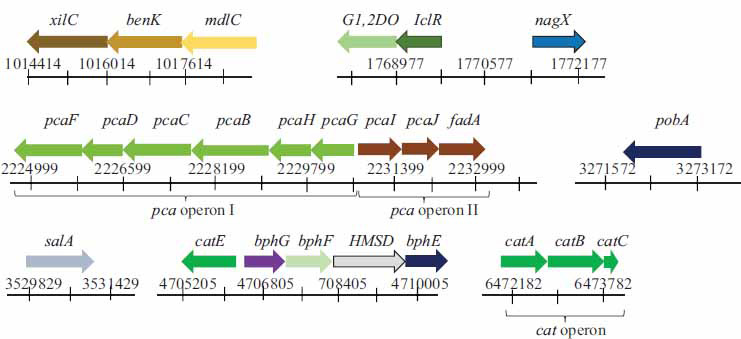
Gene organization of the mono-hydroxybenzoate degradation enzymes encoding gene cluster in the *R. opacus* KT112-7. The description of genes is presented in section 3.4.

As a result of genome analysis, the genes involved in the degradation of hydroxybenzoic acids were identified: three putative operons, *pcaGHBCDF*, *pcaIJfadA*, *catABC* and several free genes possibly participated in HBA degradation (Fig. **6**). It was found that all these genes are located on the chromosome of the strain KT112-7. Based on the analysis of translated sequences of these genes and their similarity with known sequences of enzymes involved in the degradation of aromatic compounds (Table **S6**), it is hypothesized that the identified genes encode the following enzymes: *xylC* (1458 bp) – benzaldehyde dehydrogenase, *benK* – transport protein, *mdlC* (1587 bp) – benzaldehyde decarboxylase, *G1,2DO* (1113 bp) – gentisate 1,2-dioxygenase, *iclR* – regulatory protein, *nagX* (1200 bp) – 3-hydroxybenzoate-6-hydroxylase, *pcaF* (1351 bp) – 3-oxoadipyl-CoA thiolase, *pcaD* (798 bp) – 3-oxoadipate-enol-lactonase, *pcaC* (1203 bp) – 4-carboxymuconolactone decarboxylase, *pcaB* (1353 bp) – carboxymuconate cycloisomerase, *pcaH* (714 bp) – *β-*subunit of protocatechuate 3,4-dioxygenase, *pcaG* (642 bp) – *α*-subunit of protocatechuate 3,4-dioxygenase, *pcaI* (750 bp) – *α*-subunit of 3-oxoadipate-CoA transferase, *pcaJ* (642 bp) – β-subunit of 3-oxoadipate-CoA transferase, *fadA* (745 bp) – acetyl-CoA acyltransferase, *pobA* (1179 bp) – 4-hydroxybenzoate 3-monooxygenase, *salA* (1617 bp) – salicylate hydroxylase, *catE* (1083 bp) – catechol 2,3-dioxygenase, *hmsD* (1497 bp) – 2-hydroxymuconate semialdehyde dehydrogenase, *bphG* (786 bp) – acetaldehyde dehydrogenase, *bphE* (753bp) – 2-keto-4-pentenoat hydratase; *bphF* (1059bp) – 4-hydroxy-2-oxovalerat aldolase, *catA* (843bp) – catechol 1,2-dioxygenase, *catB* (1122bp) – muconate cycloisomerases, *catC* (282bp) – muconolactone isomerase. An analysis of the deduced amino acid sequences using the GenBank database revealed that the amino acid sequences of the enzymes encoded by the identified genes involved in the degradation of hydroxybenzoic acids showed a high level of similarity (95-99%) with homologous enzymes from strains of the genus *Rhodococcus* (Table **S6**), including enzymes from known aromatic compound-degrading strains such as *R. opacus* 1CP and *R. jostii* RHA1.

**Fig. (7):**
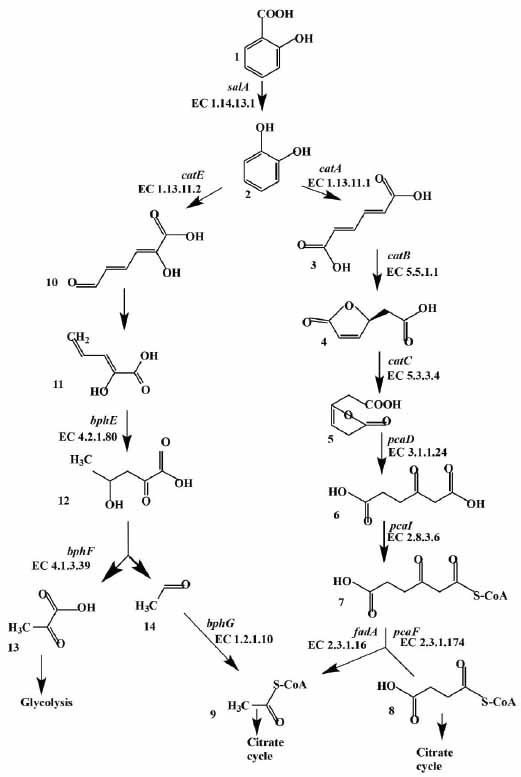
Scheme of degradation of 2-hydroxybenzoic acid by the *R. opacus* strain KT112-7: 1 – 2-hydroxybenzoate; 2 – catechol; 3 *– cis, cis*-muconate; 4 – muconolactone; 5 – 3-oxoadipate enol-lactone; 6 – 3-oxoadipate; 7 – 3-oxoadipyl-CoA; 8 – succinyl-CoA; 9 – acetyl-CoA; 10 – 2-hydroxymuconate semialdehyde; 11 – 2-oxopenta-4-enoate; 12 – 4-hydroxy-2-oxovalerate; 13 - pyruvic acid; 14 – acetaldehyde. The names of genes and numbers of enzymes catalyzing the reaction, identified during the analysis of the genome of strain KT112-7, are indicated next to the arrows.

**Fig. (8):**
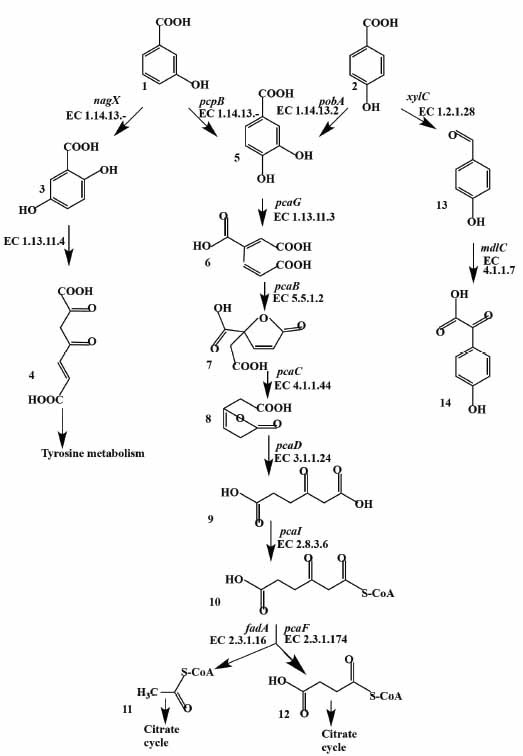
Scheme of degradation of 3-hydroxybenzoic and 4-hydroxybenzoic acids by the *R. opacus* strain KT112-7. 1 – 3-hydroxybenzoate; 2 – 4-hydroxybenzoate; 3 – gentisate; 4 – 3-maleylpyruvate; 5 – protocatechuate; 6 – *β*-carboxymuconate; 7 – 4-carboxymuconolactone; 8 – 3-oxoadipate enol-lactone; 9 – 3-oxoadipate; 10 – 3-oxoadipyl-CoA; 11 – succinyl-CoA; 12 – acetyl-CoA; 13 – 4-hydroxybenzaldehyde; 14 – 4-hydroxybenzoylformate. The names of genes and numbers of enzymes catalyzing the reaction, identified during the analysis of the genome of the strain KT112-7, are indicated next to the arrows.

These results allowed us to propose degradation pathways for mono-hydroxybenzoic acids in the strain KT112-7 (Figs. **7** and **8**).

The 2-hydroxybenzoate is firstly converted to catechol, a key intermediate in the process of 2-hydroxybenzoate metabolism. The key enzyme is salicylate hydroxylase (Figs. **6** and **7**). The presence of catechol was also confirmed experimentally (Fig. **1a**). The highest level of similarity for this enzyme was found with the homologous enzyme from the strains *R. opacus* 1CP (GenBank ANS25919.1) – 98% and *R. jostii* RHA1 (GenBank ABG96696.1) – 95% (Table **S6**). The metabolism of the formed catechol occurs through two classical pathways, mediated by catechol 1,2-dioxygenase (CatA) and catechol 2,3-dioxygenase (CatE), resulting in products that enter the tricarboxylic acid cycle and glycolysis (Fig. **7**) [81]. In this study, several genes regulating catechol degradation was identified: *catABC*, *catE*, *pcaD*, *pcaI*, *pcaF*, *fadA*, *bphEFG* (Fig. **6**). The data obtained as a result of genetic analysis confirm our assumption that strain KT112-7 is capable of using 2-hydroxybenzoic acid as a sole carbon source, expressed on the basis of the analysis of batch cultivation experiments (Fig. **2b**).

During the degradation of 3HBA, the metabolites 2,5-dihydroxybenzoic acid and 3,4-dihydroxybenzoic acid were detected (Fig. **1b**). The initial oxidation of 3HBA is catalyzed by two enzymes: 3-hydroxybenzoate monooxygenase (PcpB), which is 99% similar to the homologous penta-chlorophenol monooxygenase of the strain *R. opacus* 1CP (GenBank ANS30121.1), resulting in the formation of 3,4-dihydroxybenzoic acid (protocatechuate); and 3-hydroxybenzoate 6-hydroxylase (NagX), which is 99% similar to the 3-hydroxybenzoate 6-hydroxylase of strain *R. opacus* 1CP (GenBank ANS30049.1), resulting in the formation of 2,5-dihydroxybenzoic acid (gentisate) (Fig. **8**, Table **S6**). The further transformation of gentisate occurs through the action of gentisate 1,2-dioxygenase, with the products entering the tyrosine metabolism. The metabolism of protocatechuate proceeds through the classical enzymes of the *β*-ketoadipate pathway to compounds that enter the tricarboxylic acid cycle, which is caused by the presence of the operons *pcaGHBCDF* and *pcaIJfadA* in the genome of the strain KT112-7 (Fig. **6**).

For the degradation of 4HBA, the main metabolite identified was 3,4-dihydroxybenzoic acid (Fig. **1c**). This reaction is caused by a pobA-encoded *para*-hydroxybenzoate hydroxylase [35], which was also observed in strain KT112-7. *Para*-hydroxybenzoate hydroxylase of the strain KT112-7 is 98% similar to the homologous enzyme of strain *R. opacus* 1CP (GenBank ANS30736.1) and 95% similar to the enzyme of strain *R. jostii* RHA1 (GenBank ABG94344.1) (Fig. **8**, Table **S6**). The action of *para*-hydroxybenzoate hydroxylase transforms 4HBA into protocatechuate, which is further metabolized through the *β*-ketoadipate pathway to compounds of the central metabolism.

In addition to the above processes, strain KT112-7 probably carries another pathway of the 4HBA-biodestruction, caused by the genes xylC and mdlC, present in its genome (Figs. **6** and **8**, Table **S6**). Benzaldehyde dehydrogenase (XylC) is 99% similar to the homologous enzyme of strain *R. opacus* 1CP (GenBank ANS24764.1) and 98% similar to the enzyme of strain *R. jostii* RHA1 (GenBank ABG91840.1) (Table **S6**). If 4HBA is transformed by benzaldehyde dehydrogenase, 4-hydroxybenzaldehyde is formed. It is probable that this compound is further transformed by decarboxylase to 4-hydroxybenzoylformate. The further metabolism of this compound in the strain KT112-7 could not be determined.

In conclusion, the analysis of the metabolic profile and genetic structures of the strain KT112-7, as well as previous studies on the degradation of aromatic compounds by this strain [16], allowed us to establish the metabolic pathways for mono-hydroxylated biphenyls and mono-hydroxybenzoic acids and confirm the ability of the strain KT112-7 to completely degrade these compounds.

## CONCLUSION

The genome analysis of the *Rhodococcus opacus* KT112-7 revealed a unique set of genes responsible for the degradative activity of this strain towards substituted biphenyls and their metabolites. It was shown that the biphenyl-degrading genes located on the pRHWK1 have a high level of identity with classical *bph* genes, while the biphenyl-degrading genes located on the chromosome show a high level of similarity with naphthalene degradation genes. The genome analysis, physiological characteristics, and formation of metabolites during the degradation of mono-hydroxybiphenyls revealed that the strain KT112-7 degrades mono-hydroxylated biphenyls through the classical pathway involving dioxygenases and further metabolizes the formed basal metabolic compounds of the cell. Thus, the strain KT112-7 holds promise for nature-inspired biotechnological applications aimed at the degradation of biphenyl and its derivatives.

## STUDY LIMITATIONS

This work presents an analysis of the biodegradation potential of strain KT112-7 toward hydroxylated biphenyls based on metabolite data and the whole-genome sequence. However, to confirm the proposed metabolic pathways, gene expression studies are necessary.

## Figures and Tables

**Table 1:** Kinetic parameters of the strain KT112-7.

**Substrate**	**Specific Growth Rate, Day^-1^**	**Specific Degradation Rate, Day^-1^**
2НО-biphenyl	0.168	0.087
3НО-biphenyl	1.289	0.561
4НО-biphenyl	1.203	0.311
2-hydroxybenzoic acid	0.044	0.037
3-hydroxybenzoic acid	0.193	1.741
4-hydroxybenzoic acid	0.777	1.710

## Data Availability

The authors confirm that the data supporting the findings of this research are available within the article.

## LIST OF ABBREVIATIONS

ANI: Average Nucleotide Identity
KEGG: Kyoto Encyclopedia of Genes and Genomes
PCBs: Polychlorinated Biphenyls
PDB: Protein Data Bank
