# Supplementary material for: An Assessment of the Degradation Potential and Genomic Insights Towards Hydroxylated Biphenyls by Rhodococcus opacus Strain KT112-7
Source: Curr Genomics. 2024 Aug 21;26(5):341–58. doi: 10.2174/0113892029319746240812051356 (PMC12728593; doi:10.2174/0113892029319746240812051356)
Supplement: Supplementary file 1 [file CG-26-5-341_SD1.pdf]

Supplementary Material

An Assessment of the Degradation Potential and Genomic Insights Towards Hydroxylated Biphenyls by *Rhodococcus opacus* Strain KT112-7

Darya Egorova[1,*], Bjorn Olsson[2], Tatyana Kir'yanova[1] and Elena Plotnikova[1]

[1]Laboratory of Microbiology of Technogenic Ecosystems, Institute of Ecology and Genetics of Microorganism, UB RAS, Perm, Russia; [2]School of Biosciences, University of Skövde, Skövde, Sweden

Table 1S. Genome features of the *Rhodococcus opacus* KT112-7.

Feature	Chromosome	Megaplasmid pRHWK1	Megaplasmid pRHWK2
Genom size (bp)	7587912	281912	130937
GC content (%)	67.5	64.3	64.1
RNA genes	53	0	0
Protein coding sequences (CDS)	7445	326	160

Table 2S. Comparison of transcribed gene sequences for the degradation of biphenyl and its derivatives obtained during the analysis of the genome of the strain KT112-7 with homologous sequences from the GenBank database.

Enzyme, Number in the EC Classification, Localization of the Gene Encoding it	The Closest Homologous Sequences from GenBank, GenBank Number, Strains Degrading Aromatic Hydrocarbons	Identity, %
Biphenyl dioxygenase alpha subunit (BphA1), **EC 1.14.12.18**, chromosome	NarC, BAH47216.1, *Rhodococcus opacus* B4<br>NidD, AAD25398.1, *Rhodococcus* sp. I24<br>NarC, AAR05109.1, *Rhodococcus* sp. P400	99<br>99<br>99
Biphenyl dioxygenase beta subunit (BphA2), **EC 1.14.12.18**, chromosome	NarAb, ADM94822.1, *Rhodococcus* sp. G10<br>NarAb, BAH47213.1, *Rhodococcus opacus* B4<br>NarAb, AAR05107.1, *Rhodococcus* sp. P400	100<br>99<br>99
2,3-dihydroxy-2,3-dihydro-biphenyl dehydro-genase (BphB), **EC 1.3.1.87**, chromosome	NarB, BAH47215.1, *Rhodococcus opacus* B4<br>NarB, AQW45619.1, *Rhodococcus ruber* OA1<br>HcaB, QSE72234.1, *Rhodococcus* sp. PSBB049	99<br>99<br>99
2,3-dihydroxybiphenyl 1,2-dioxygenase (BphC), **EC 1.13.11.39**, chromosome	HsaC2, ANS26769.1, *Rhodococcus opacus* 1CP<br>Extradiol dioxygenase, type I, ABG97580.1, *Rhodococcus jostii* RHA1<br>Extradiol dioxygenase, BAH54111.1, *Rhodococcus opacus* B4	100<br>96<br>92
2,3-dihydroxybiphenyl 1,2-dioxygenase (BphC), **EC 1.13.11.39**, chromosome	BphC, ANS24889.1, *Rhodococcus opacus* 1CP<br>BphC, QDQ89556.1, *Rhodococcus* sp. WB9<br>Metapyrocatechase 2, AHK27384.1, *Rhodococcus opacus* PD630	99<br>99<br>99
2-hydroxy-6-oxo-6-phenylhexa-2,4-dienoate hydrolase (BphD), **EC 3.7.1.-**, chromosome	Hydrolase, ANS28558.1, *Rhodococcus opacus* 1CP<br>Hydrolase, ABG92156.1, *Rhodococcus jostii* RHA1<br>Hydrolase,AII03552.1, *Rhodococcus opacus* R7	100<br>97<br>95

2-hydroxy-6-oxo-6-phenylhexa-2,4-dienoate hydrolase (BphD), **EC 3.7.1.-,** chromosome	alpha/beta hydrolase,AUS35655.1, *Rhodococcus qingshengii* djl-6-2 pDJL1<br>alpha/beta hydrolase, ANQ76140.1, *Rhodococcus* sp. 008	98<br>98
2-keto-4-pentenoate hydratase (BphH(E)), **EC 4.2.1.80**, chromosome	mhpD2, ANS26765.1, *Rhodococcus opacus* 1CP<br>2-keto-4-pentenoate hydratase,QDQ91340.1, Rhodococcus sp. WB9<br>BphH(E), ABG97576.1, *Rhodococcus jostii* RHA1	99<br>97<br>95
Acetaldehyde dehydrogenase (BphJ(G)), **EC 1.2.1.10**, chromosome	Acetaldehyde dehydrogenase, WP_283290080.1, *Rhodococcus* sp. IEGM 1351<br>Acetaldehyde dehydrogenase 4, QOS4F9.1, *Rhodococcus jostii* RHA1<br>Acetaldehyde dehydrogenase, WP_192815083.1, *Rhodococcus* sp. DK17	99<br>99<br>99
4-hydroxy-2-oxovalerate aldolase (BphI(F)), **EC 4.1.3.39**, chromosome	4-hydroxy-2-oxovalerate aldolase 4, ANS26767.1, *Rhodococcus opacus* 1CP<br>4-hydroxy-2-oxovalerate aldolase, QDQ91342.1, Rhodococcus sp. WB9<br>4-hydroxy-2-oxovalerate aldolase, ABG97578.1, *Rhodococcus jostii* RHA1	99<br>99<br>97
biphenyl 2,3-dioxygenase alpha subunit (BphA1), **EC 1.14.12.18**, pRHWK1	BphA1, ABG99107.1, *Rhodococcus jostii* RHA1<br>BnzA1, BAD95523.1, *Rhodococcus opacus* B4<br>toluene-inducible dioxygenase, AAL61663.2<br>*Rhodococcus aetherivorans* I24	100<br>100<br>99
Biphenyl 2,3-dioxygenase subunit beta (BphA2), **EC 1.14.12.18**,pRHWK1	BphA2,ABG99106.1, *Rhodococcus jostii* RHA1<br>BnzA2, BAD95524.2, *Rhodococcus opacus* B4<br>iron sulfur protein small subunit, AAL61664.1, *Rhodococcus aetherivorans* I24	100<br>100<br>97
Biphenyl 2,3-dioxygenase, ferredoxin (BphA3), pRHWK1	IpbA3, AAP74040.1, *Rhodococcus erythropolis* BD2<br>BtfA3, BAQ00538.1, *Rhodococcus* sp. 065240<br>BnzA3, BAD95525.1, *Rhodococcus opacus* B4	100<br>100<br>99
Biphenyl 2,3-dioxygenase, ferredoxin reductase component (BphA4), **EC 1.18.1.3**, pRHWK1	BnzA4, BAD95526.2, *Rhodococcus opacus* B4<br>IpbA4, AAP74041.1, *Rhodococcus erythropolis* BD2<br>BphAc, ABG99105.1, *Rhodococcus jostii* RHA1	100<br>100<br>91
2,3-dihydroxy-2,3-dihydro-biphenyl dehydrogenase (BphB), **EC 1.3.1.87**, pRHWK1	BtfB, BAQ00541.1, *Rhodococcus* sp. 065240<br>BnzB, BAD95528.1, *Rhodococcus opacus* B4<br>BphB, ABG99101.1, *Rhodococcus jostii* RHA1	100<br>99<br>99
2,3-dihydroxybiphenyl 1,2-dioxygenase (BphC), EC **1.13.11.39**,pRHWK1	BphC, BAA06872.1, *Rhodococcus jostii* RHA1<br>BnzC, BAD95527.2, *Rhodococcus opacus* B4<br>IpbC, AAP74042.1, *Rhodococcus erythropolis* BD2	100<br>100<br>100
2-keto-4-pentenoate hydratase (BphH(E)), EC **4.2.1.80**, pRHWK1	BphE3, ABG99132.1, *Rhodococcus jostii* RHA1<br>2-hydroxypenta-2,4-dienoate hydratase, BAH47233.1, *Rhodococcus opacus* B4<br>MhpD4, ANS28948.1, *Rhodococcus opacus* 1CP	92<br>95<br>97
biphenyl 2,3-dioxygenase alpha subunit (BphA1), **EC 1.14.12.18**, pRHWK2	NarC, BAH47216.1, *Rhodococcus opacus* B4 plasmid pROB02<br>Aldolase, BAE53379.1, *Rhodococcus opacus* TKN14<br>NidD, AAD25398.1, *Rhodococcus* sp. I24	99<br>99<br>99
Biphenyl 2,3-dioxygenase subunit beta (BphA2), EC **1.14.12.18**,pRHWK2	narAb, ADM94822.1, *Rhodococcus* sp. G10<br>narAb, BAH47213.1, *Rhodococcus opacus* B4 plasmid pROB02<br>nidB, BAE53377.1, *Rhodococcus opacus* TKN14	100<br>99<br>99

Table 3S. Amino acid sequences selected from the PDB with the highest level of similarity for biphenyl 2,3-dioxygenase the strain KT112-7.

Description	Max Score	Total Score	Query Cover	E value	Per. Ident	Acc. Len	Accession
Biphenyl dioxygenase (BphA1A2) derived from *Rhodococcus* sp. strain RHA1 [*Rhodococcus jostii* RHA1]	952	952	100%	0.0	98.70%	460	1ULI_A
Crystal structure of toluene 2,3-dioxygenase [*Pseudomonas putida*]	746	746	95%	0.0	77.68%	450	3EN1_A
Crystal structure of II9 variant of biphenyl dioxygenase from *Burkholderia xenovorans* LB400 [*Paraburkholderia xenovorans* LB400]	659	659	93%	0.0	69.32%	459	5AEU_A
Crystal structure of biphenyl dioxygenase variant RR41 (BPDO-RR41) [*Paraburkholderia xenovorans* LB400]	656	656	93%	0.0	68.86%	459	2YFI_A
Crystal structure of P4 variant of biphenyl dioxygenase from *Burkholderia xenovorans* LB400 in complex with 2,6 di chlorobiphenyl [*Paraburkholderia xenovorans* LB400]	654	654	93%	0.0	68.64%	459	2XSH_A
Crystal structure of biphenyl dioxygenase from *Burkholderia xenovorans* LB400 [*Paraburkholderia xenovorans* LB400]	653	653	93%	0.0	68.64%	459	2XR8_A
Cumene dioxygenase (cumA1A2) from *Pseudomonas fluorescens* IP01 [*Pseudomonas fluorescens*]	636	636	94%	0.0	66.59%	459	1WQL_A
Crystal structure of the biphenyl dioxygenase in complex with biphenyl from *Comamonas testosteroni* sp. strain B-356 [*Comamonas testosteroni*]	640	640	97%	0.0	65.64%	457	3GZX_A

Table 4S. Parameters of 3D models of the *α*-subunit of biphenyl 2,3-dioxygenase the strain KT112-7.

```
>> Summary of successfully produced models:
Filename                          molpdf     DOPE score    GA341 score
----------------------------------------------------------------------
33.B99990001.pdb              2876.91382   -51376.98047        1.00000
33.B99990002.pdb              2878.96558   -51696.81250        1.00000
33.B99990003.pdb              3001.39795   -51464.85156        1.00000
33.B99990004.pdb              2906.20312   -51636.73438        1.00000
33.B99990005.pdb              2846.77612   -51610.49219        1.00000
```

Table 5S. Results of molecular docking of monohydroxylated biphenyls and α subunit biphenyl 2,3-dioxygenase of the strain KT112-7.

Monohydroxybiphenyl	Contact Area *	Number of Clusters, pcs	Number of Positions of Hydroxybiphenyl Molecules, pcs	Affinity Index, ΔG, J/mol**
2HO-biphenyl	Orange	2	16	-6.581<br>-6.892
	Green	4	54	-5.695<br>-5.812<br>-5.896<br>-6.011
	Yellow	8	32	-5.606<br>-5.683<br>-5.769<br>-5.816<br>-5.818<br>-5.864<br>-5.904<br>-6.040
	Blue	1	8	-5.658
3HO-biphenyl	Orange	0	0	-
	Green	10	96	-5.632<br>-5.645<br>-5.654<br>-5.720<br>-5.742<br>-5.815<br>-5.819<br>-5.880<br>-5.887<br>-6.072
	Yellow	7	32	-5.526<br>-5.686<br>-5.841<br>-5.963<br>-5.998<br>-6.062<br>-6.095
	Blue	1	8	-6.208

4HO-biphenyl	Orange	0	0	-
	Green	5	43	-5.615<br>-5.762<br>-5.864<br>-6.019<br>-6.155
	Yellow	6	24	-5.248<br>-5.745<br>-5.897<br>-5.919<br>-5.962<br>-5.977
	Blue	1	8	-5.856

Note. * - color corresponds to the color of the area in Figure 5, ** - average ΔG value for each cluster is indicated

Table 6S. Comparison of transcribed gene sequences for the degradation of hydroxybenzoic acids and its derivatives obtained during the analysis of the genome of the strain KT112-7 with homologous sequences from the GenBank database.

**Enzyme, Number in the EC Classification, Localization of the Gene Encoding it**	**The Closest Homologous Sequences from GenBank, GenBank Number, Strains Degrading Aromatic Hydrocarbons**	**Identity, %**
Salicylate hydroxylase/ 2-hydroxybenzoate hydroxylase, **EC 1.14.13.1,** chromosome	FAD-dependent monooxygenase, QQZ12995.1, *Rhodococcus* sp. 21391<br>Monooxygenase, ANS25919.1, *Rhodococcus opacus* 1CP<br>Monooxygenase, ABG96696.1, *Rhodococcus jostii* RHA1	99<br>98<br>95
4-hydroxybenzoate hydroxylase, **EC 1.14.13.2**, chromosome	PobA, AHF21002.1, *Rhodococcus opacus* 557<br>PobA, ANS30736.1, *Rhodococcus opacus* 1CP<br>4-hydroxybenzoate 3-monooxygenase, ABG94344.1, *Rhodococcus jostii* RHA1	99<br>98<br>97
FAD-binding monooxygenase/3-hydroxybenzoate hydroxylase, **EC 1.14.13.-,** chromosome	Pentachlorophenol monooxygenase, ANS30121.1, *Rhodococcus opacus* 1CP<br>FAD-dependent oxidoreductase, QDQ94206.1, *Rhodococcus* sp. WB9<br>*Pentachlorophenol monooxygenase*, ABG93750.1, *Rhodococcus jostii* RHA1	99<br>99<br>93
n-hydroxybenzoate hydroxylase/3-hydroxybenzoate hydroxylase, **EC 1.14.13.-,** chromosome	FAD-dependent monooxygenase, QQZ15601.1, *Rhodococcus* sp. 21391<br>3-hydroxybenzoate 6-hydroxylase, QDQ94141.1, *Rhodococcus* sp. WB9<br>3-hydroxybenzoate 6-hydroxylase, ANS30049.1, *Rhodococcus opacus* 1CP	99<br>99<br>99
Protocatechuate 3,4-dioxygenase alpha chain, **EC 1.13.11.3**, chromosome	PcaG, ANS29566.1, *Rhodococcus opacus* 1CP<br>PcaG, QDQ93627.1, *Rhodococcus* sp. WB9<br>Protocatechuate 3,4-dioxygenase alpha chain, AHK32654.1, *Rhodococcus opacus* PD630	99<br>99<br>97

Protocatechuate 3,4-dioxygenase beta chain, **EC 1.13.11.3**, chromosome	PcaH, QDQ93627.1, *Rhodococcus* sp. WB9<br>PcaH, ANS29565.1, *Rhodococcus opacus* 1CP<br>pcaH, ABG93159.1, *Rhodococcus jostii* RHA1	99<br>99<br>97
Carboxymuconat cycloisomerase, **EC 3.5.1.2**, chromosome	PcaB, ELB89551.1, *Rhodococcus wratislaviensis* IFP2016,<br>PcaB, NHU43320.1, *Rhodococcus* sp. A14<br>PcaB, EID79918.1, *Rhodococcus opacus* RKJ300	100<br><br>100<br>100
Carboxy-muconolacton decarboxylase, **EC 4.1.1.44**, chromosome	PcaC, ELB89552.1, *Rhodococcus wratislaviensis* IFP2016,<br>PcaC, NHU43319.1, *Rhodococcus* sp. A14<br>PcaC, EID79919.1, *Rhodococcus opacus* RKJ300	100<br><br>100<br>100
3-oxoadipat enol-lactonase, **EC 3.1.1.24**, chromosome	PcaD, ELB89171.1, *Rhodococcus wratislaviensis* IFP2016,<br>PcaD, NHU46033.1, *Rhodococcus* sp. A14<br>PcaD, EKT79318.1, *Rhodococcus opacus* M213	100<br><br>100<br>100
3-oxoadipil-CoA thiolase, **EC 2.3.1.174**, chromosome	PcaF, GAF 42365.1, *Rhodococcus wratislaviensis* NBRC 100605<br>PcaF, CAG7581393.1, *Rhodococcus opacus* DSM43205<br>PcaF REE76879.1, *Rhodococcus wratislaviensis* WS3308	100<br><br>99<br>99
3-oxoadipat-succinyl-CoA transferase alpha chain, **EC 2.8.3.6**, chromosome	PcaI, ELB89166.1, *Rhodococcus wratislaviensis* IFP2016<br>PcaI, NDV 05409.1, *Rhodococcus* sp. IEGM 248<br>PcaI, NHU 46037.1, *Rhodococcus* sp. A14	100<br><br>100<br>100
3-oxoadipat-succinyl-CoA transferase beta chain, **EC 2.8.3.6**, chromosome	PcaJ, ELB 90265.1, *Rhodococcus wratislaviensis* IFP2016<br>PcaJ, NHU 45591.1, *Rhodococcus* sp. A14<br>PcaJ, KXF 54969.1, *Rhodococcus* sp. SC4	100<br><br>100<br>100
Acetil-CoA-acil-transferase, **EC 2.3.1.16**, chromosome	FadA, ANS29570.1, *Rhodococcus opacus* 1CP<br>FadA, ABG 93164.1, *Rhodococcus jostii* RHA1<br>FadA, MBP 2208086.1, *Rhodococcus opacus* WS 3313	100<br>99<br>99
Catechol 1,2-dioxigenase, **EC 1.13.11.1**, chromosome	CatA, ELB 86550.1, *Rhodococcus wratislaviensis* IFP2016<br>CatA, NHU 43321.1, *Rhodococcus* sp. A14<br>CatA, MDI 9937715.1, *Rhodococcus* sp. IEGM 1351	100<br><br>100<br>100
Muconate cycloisomerases, **EC 5.5.1.1**, chromosome	CatB, ELB 86551.1, *Rhodococcus wratislaviensis* IFP2016<br>CatB, EID 79918.1, *Rhodococcus opacus* RKJ 300<br>CatB, QQZ15125.1, *Rhodococcus* sp. 21391	100<br><br>99<br>99
Muconolacton isomerase, **EC 5.3.3.4**, chromosome	CatC, ELB 86552.1, *Rhodococcus wratislaviensis* IFP2016<br>CatC, NHU43319.1, *Rhodococcus* sp. A14<br>CatC, EID 79919.1, *Rhodococcus opacus* RKJ 300	100<br><br>100<br>100

Catechol 2,3-dioxigenase, **EC 1.13.11.2**, chromosome	CatE, ELB 88197.1, *Rhodococcus wratislaviensis* IFP2016<br>CatE, WP_271213298.1, *Rhodococcus wratislaviensis* VKM Ac-2782<br>CatE, EKT 79780.1, *Rhodococcus opacus* M13	100<br>99<br>99
Gentisate 1,2-dioxigenase, **EC 1.13.11.4**, chromosome	G1,2DO, UNN00157.1, *Rhodococcus opacus* S8<br>G1,2DO, CAG 7583476.1, *Rhodococcus opacus* DSM 43205<br>G1,2DO, MDI9935522.1, *Rhodococcus* sp. IEGM 1351	100<br>99<br>99
Benzaldehyde-dehidrogenase, **EC 1.2.1.28**, chromosome	XylC, EKT79777.1, *Rhodococcus opacus* M213<br>XylC, MDI9939923.1, *Rhodococcus* sp. IEGM 1351<br>XylC, QDQ89598.1, *Rhodococcus* sp. WB9	100<br>100<br>100
Benzoate MFC transporter BenK, chromosome	BenK, ELB87238.1, *Rhodococcus wratislaviensis* IFP2016<br>GenK, CAG7585576.1, *Rhodococcus opacus* DSM43205<br>BenK, MBA8961962.1, *Rhodococcus opacus* DSM 44240	100<br>100<br>99
Benzaldehyde decarboxylase, **EC 4.1.1.7**, chromosome	MdlC, ELB89171.1, *Rhodococcus wratislaviensis* IFP2016<br>MdlC, NHU46033.1, *Rhodococcus* sp. A14<br>MdlC, EKT79318.1, *Rhodococcus opacus* M213	100<br>100<br>100

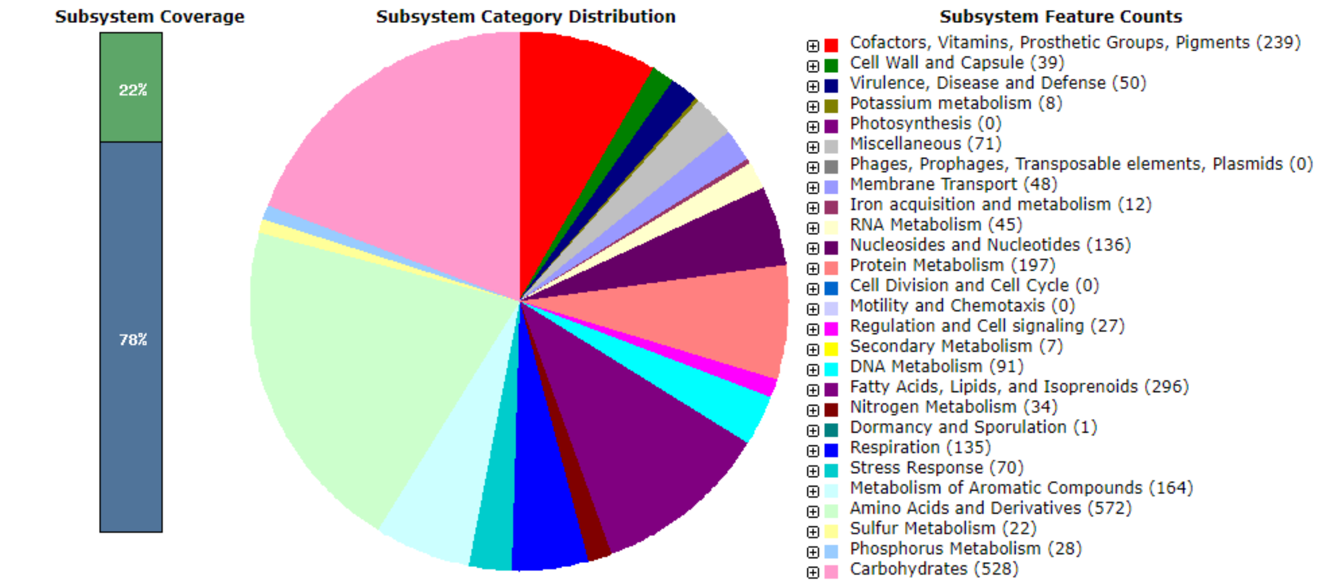

(a)

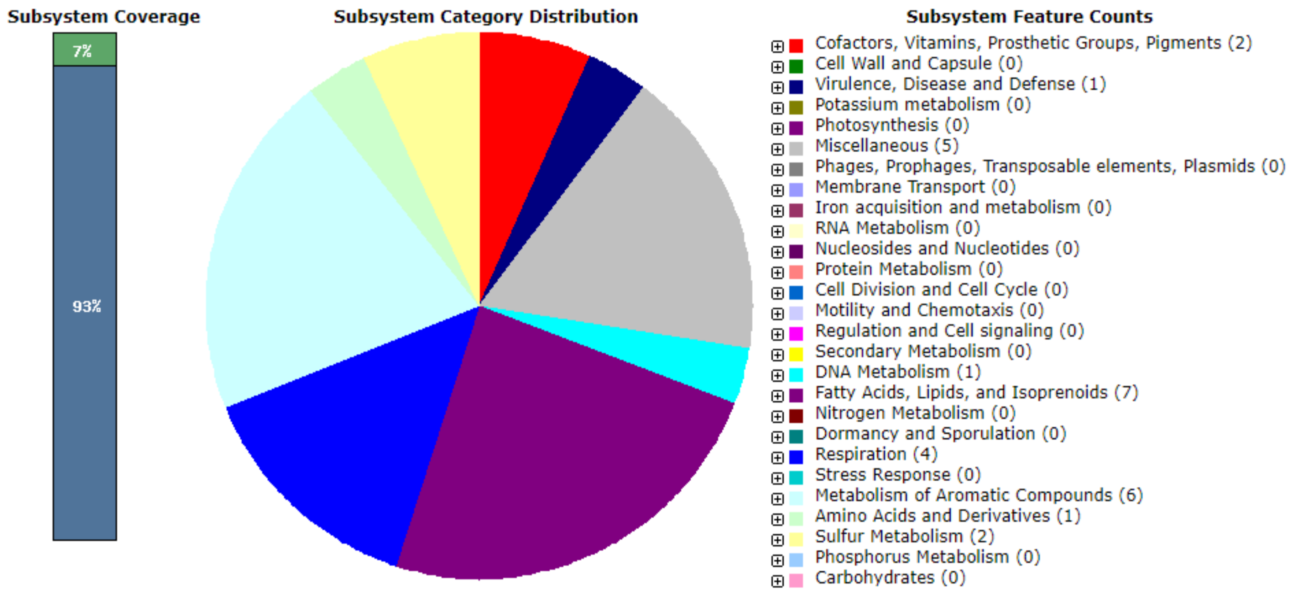

(b)

Fig. (1S). Functional subsystem analysis of annotated genome of the *Rhodococcus opacus* KT112-7: **a** – chromosome, **b** – megaplasmid pRHWK1. Functional categories are presented on the right part of the figures with blanket, which shows number of genes. The subsystem coverage bar charts to the left in each figure show the percentage of genes that could be linked to a subsystem (22% for the chromosome, 7% for the plasmid).

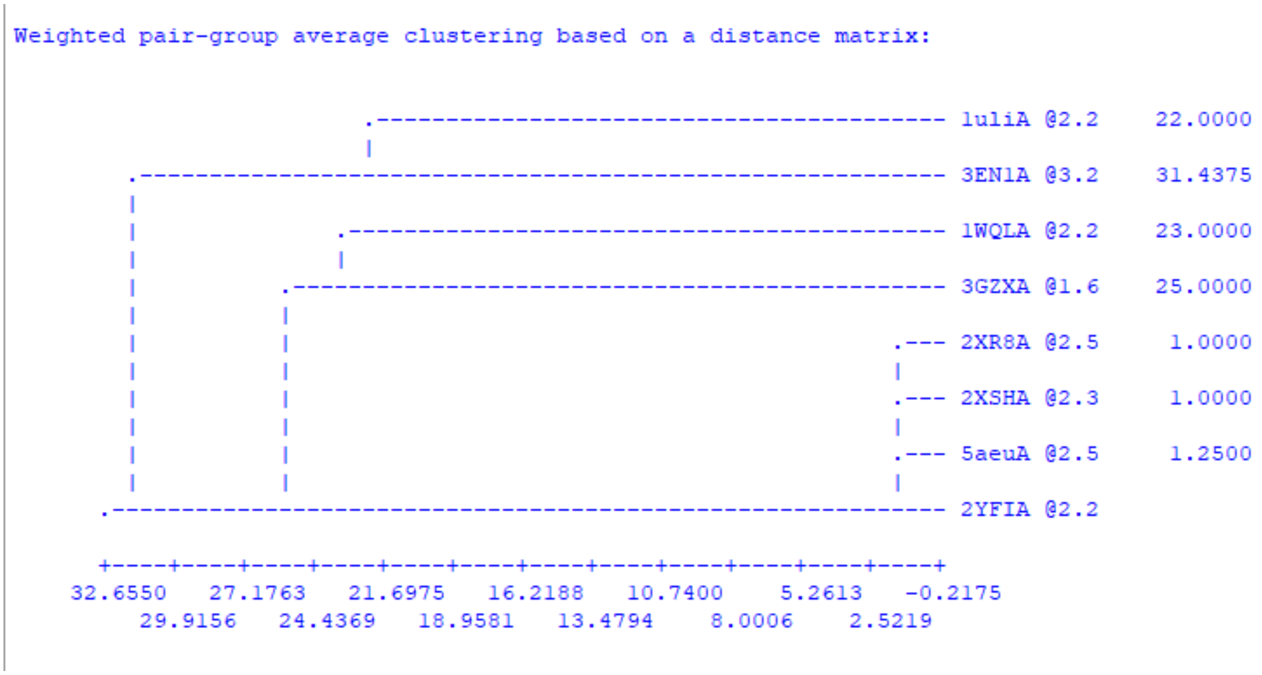

Fig. (2S). Dendrogram of selected amino acid sequences of *α*-subunits of biphenyl 2,3-dioxygenase (indicating affinity and crystallography index).
